# Clustering Vector Autoregressive Models: Capturing Qualitative Differences in Within-Person Dynamics

**DOI:** 10.3389/fpsyg.2016.01540

**Published:** 2016-10-07

**Authors:** Kirsten Bulteel, Francis Tuerlinckx, Annette Brose, Eva Ceulemans

**Affiliations:** ^1^Faculty of Psychology and Educational Sciences, KU LeuvenLeuven, Belgium; ^2^Institute for Psychology, Humboldt University BerlinBerlin, Germany

**Keywords:** time series analysis, cluster analysis, vector autoregressive modeling, partitioning, individual differences

## Abstract

In psychology, studying multivariate dynamical processes within a person is gaining ground. An increasingly often used method is vector autoregressive (VAR) modeling, in which each variable is regressed on all variables (including itself) at the previous time points. This approach reveals the temporal dynamics of a system of related variables across time. A follow-up question is how to analyze data of multiple persons in order to grasp similarities and individual differences in within-person dynamics. We focus on the case where these differences are qualitative in nature, implying that subgroups of persons can be identified. We present a method that clusters persons according to their VAR regression weights, and simultaneously fits a shared VAR model to all persons within a cluster. The performance of the algorithm is evaluated in a simulation study. Moreover, the method is illustrated by applying it to multivariate time series data on depression-related symptoms of young women.

## Introduction

In psychology, studying multivariate within-person processes across time is gaining ground (Molenaar, 2004; Hamaker, 2012; Hamaker et al., 2015). For instance, in emotion psychology, Pe and Kuppens (2012) studied the dynamical interplay of emotions in daily life. More specifically, they investigated if the experience of a particular emotion at a specific occasion increased or decreased the intensity of another emotion at the next occasion. As another example, Snippe et al. (2015) analyzed the person-specific temporal relations between mindfulness, repetitive thinking, and depressive symptoms during mindfulness-based treatment. Finally, Rosmalen et al. (2012) inspected the dynamics between depression level and physical activity after a myocardial infarction.

Individual differences in these within-person dynamics are often present. In many cases, it is plausible for empirical or theoretical reasons that these differences are qualitative in nature. Empirical findings indeed regularly suggest that subgroups of persons exist that are characterized by similar dynamics. For example, in the study of Rosmalen et al. (2012) mentioned above, past activity level predicted the current depression score for two of the four participants, whereas the temporal direction of this relation was reversed for another participant and no significant effects were present for the final participant. Similar patterns of individual differences were reported by Pe and Kuppens (2012) and Snippe et al. (2015). In other research areas, theoretical arguments point to qualitative differences as well, but empirical evidence is yet missing. For example, attachment theory conjectures that qualitatively different patterns of attachment can be distinguished: Secure attachment, avoidant attachment, and anxious attachment (Bowbly, 1969; Ainsworth et al., 1978). The within-person dynamics of attachment-related behaviors (e.g., do children react to stressful situations by using mother as a safe haven or do they try to solve the problem on their own because they don't expect help from mother?) should differ accordingly across persons. Another example can be found in the literature on eating disorders, where anorexia nervosa, bulimia nervosa, and binge eating disorder, are presumably associated with different temporal dynamics (e.g., do patients react to body dissatisfaction by avoiding to eat or by binging?; Stice, 2002).

An important data-analytical question is then how to parsimoniously reveal these qualitative differences in within-person dynamics. In general, qualitative differences are often revealed by clustering techniques (Gan et al., 2007). To date, however, clusterwise extensions of models capturing the within-person dynamics of multiple variables are not readily available^1^. In this paper, we will present one solution to this problem, related to recent autoregressive (AR) based clustering approaches (e.g., Liao, 2005; Frühwirth-Schnatter and Kaufmann, 2008; D'Urso et al., 2013, 2015), which focus on the dynamics within single variables: We propose a clusterwise extension of the most popular time series model for studying multivariate dynamical processes, namely vector autoregressive (VAR) models (e.g., Schmitz and Skinner, 1993; Wild et al., 2010; Bos et al., 2012; Rosmalen et al., 2012; Bringmann et al., 2013; van Gils et al., 2014; Wichers, 2014; Pe et al., 2015; Snippe et al., 2015; van der Krieke et al., 2015).

In a VAR model, each variable is regressed on all other variables and itself at previous time points. Contemporaneous (i.e., at the same time point) relations between the variables are dealt with by allowing instantaneous correlations between the respective residuals (as discussed in the textbook of Hamilton, 1994; Lütkepohl, 2005). In contrast, the structural VAR (SVAR) model, less often used in psychology, adds coefficients to capture the direction and the size of contemporaneous relations. However, in order to identify a SVAR model, theory is needed to specify a priori which unidirectional contemporaneous relations are present (Lütkepohl, 2005). An advantage of VAR is thus that no such restrictions need to be imposed. The price to pay is that the coefficients are not directly interpretable (see Brandt and Williams, 2007). If the goal is to make predictions about future points in time based on past and present measurements (i.e., forecasting), this is however not an issue.

The aim of this paper is thus to develop a method to classify persons based on their temporal dependencies drawing on the VAR methodology. This clusterwise VAR model groups persons according to their VAR coefficients, and simultaneously fits a shared VAR model to all persons within a cluster. Clusters will then consist of persons with similar dynamical processes.

The remainder of the paper is organized as follows. In the next section, we shortly recapitulate the theory of VAR models, followed by the introduction of the clusterwise VAR model. In addition, the section contains a description of the algorithm and proposes a model selection procedure. In the third section, the performance of the algorithm is evaluated in a simulation study. Then, the model is illustrated with an application to time series data on depression-related symptoms of young women. To conclude, directions for future research are addressed.

## Clusterwise VAR(1) modeling

### The VAR(1) model

#### Model

VAR modeling was proposed to analyze *M*-variate time series data of length *T* (Hamilton, 1994; Lütkepohl, 2005). The model consists of a set of equations in which each of the *M* variables is separately regressed on all *M* variables (including itself) at previous occasions. As is mostly done in psychological research (e.g., Schmitz and Skinner, 1993; Wild et al., 2010; Bringmann et al., 2013; Pe et al., 2015; Krone et al., 2016; Schuurman et al., 2016), we will focus on a model predicting the score of the variables at time *t* based on the measurements at time point *t*-1, that is, a VAR model of order 1. Extending the model to higher orders is straightforward, however (Hamilton, 1994; Lütkepohl, 2005). The model formula of the VAR(1) model reads as follows:

(1) $$\begin{array}{l} {\text{y}}_{t}\;=\;\text{c}\;+\;\Phi {\text{y}}_{t\;-\;1}\;+\;{\text{u}}_{t} \end{array}$$

where the *M* × 1 vectors **y**_*t*_ and **y**_*t*__−1_ represent the values of the variables at time points *t* and *t*-1, respectively, the *M* × 1 vector **c** holds the intercepts, the *M* × *M* matrix **Φ** contains the VAR(1) regression slopes, and the *M* × 1 vector **u**_*t*_ holds the innovations at time *t*. The innovations are dynamical residuals, and they capture the part of the variable that cannot be predicted based on the scores at the previous time point.

The VAR(1) model comes with three assumptions. A first assumption is that the intervals between consecutive measurements are of equal length. Second, the innovations are assumed to follow a normal distribution with a zero mean vector and a covariance matrix **Σ**; violations of this assumption can be tackled by using a mixture distribution instead (see Fong et al., 2007). The innovations can thus be correlated at the same time point, but not across time points. Further, the time series are assumed to be stationary (i.e., the joint distribution is time invariant; Lütkepohl, 2005). This implies that the eigenvalues of **Φ** should have a modulus smaller than 1 (Lütkepohl, 2005).

VAR(1) models can be used for forecasting (Lütkepohl, 2005)^2^. Specifically, forecasts can be computed for the time points following a particular set of variable scores (i.e., a specific state of a person). In other words, given our VAR(1) parameters and certain scores for each of the variables, we can investigate how each variable would evolve assuming that there are no innovations. Think for instance of the following hypothetical example. Two persons have the same high scores on the same set of variables, but their VAR(1) slopes strongly differ, with the slopes of the first person being closer to zero than those of the second person. Now, we can inspect whether the predictions for each variable differ across those persons. The variable scores of the first person will probably quickly return to their mean value. The variable scores of the second person only slightly decrease because of the VAR(1) slopes and therefore also impact the next observations. Thus, the variables only return to their mean value after a while. Over and above this, the percentage of explained variance *R*^2^ for each variable gives an indication of the extent to which this variable can be predicted on the basis of the variables at the previous point. For the hypothetical example, the past scores of the second person predict the future observations to a greater extent than the past scores of the first person, leading to a larger *R*^2^. Both approaches—forecasting and *R*^2^—will be shown in the application below.

#### Data analysis

Various procedures including least squares (LS) estimation methods, Yule-Walker estimation, and maximum likelihood (ML) estimation, are available to estimate the parameters of a VAR model (see e.g., Hamilton, 1994; Lütkepohl, 2005). LS and ML estimators yield identical estimates (Lütkepohl, 2005). Yule-Walker estimators have the same asymptotic properties, but might be less optimal in small samples (Lütkepohl, 2005). We will only discuss multivariate LS as the clusterwise extension we will present is based on this estimation procedure. Following Lütkepohl (2005), by defining

(2) $$\begin{array}{l} \text{Y}\equiv \left({\text{y}}_{2},\ldots ,{\text{y}}_{T}\right), \end{array}$$

(3) $$\begin{array}{l} \text{B}\equiv \left(\text{c},\Phi \right), \end{array}$$

and

(4) $$\begin{array}{l} \text{Z}\equiv \left(\left[\begin{array}{c} 1\\ {\text{y}}_{1} \end{array}\right],\ldots ,\left[\begin{array}{c} 1\\ {\text{y}}_{T-1} \end{array}\right]\right), \end{array}$$

the VAR(1) model can be rewritten as follows:

(5) $$\begin{array}{l} \text{Y}=\text{B}\text{Z}\;+\;\text{U}. \end{array}$$

The VAR(1) coefficients can then be obtained by solving the following closed-form expression:

(6) $$\hat{B}=YZ\prime {\left(ZZ\prime \right)}^{-\text{1}}.$$

This estimation step is equivalent to conducting an ordinary LS estimation for each equation separately (Lütkepohl, 2005). A sufficiently large number of time points are required to obtain good estimates (typically larger than 50; see e.g., Wild et al., 2010; Rosmalen et al., 2012; Krone et al., 2016).

### The clusterwise VAR(1) model

#### Model

In this paper, we propose a clusterwise extension of the VAR(1) model. Starting from the key idea behind clusterwise linear regression (Späth, 1979, 1982; DeSarbo et al., 1989; Brusco et al., 2008), we will cluster persons according to their VAR(1) regression weights [i.e., each person *i* (*i* = 1, …, *I*) is assigned to one particular cluster *k* (*k* = 1, …, *K*)], and simultaneously, fit a shared VAR(1) model to all persons within a cluster. In terms of the taxonomy of Liao (2005), who distinguishes clustering time series based on (a) the raw data, (b) features extracted from the data, and (c) model parameters, our clustering approach is based on model parameters to ensure that we identify groups of persons with similar time dynamics.

The model formula is the following:

(7) $$\begin{array}{r} {\text{y}}_{it}=\sum_{k\;=\;1}^{K}{\text{p}}_{ik}\left({\text{c}}_{k}\;+\;\Phi_{k}{\text{y}}_{i,t-1}\;+\;{\text{u}}_{kt}\right). \end{array}$$

The *M* × 1 vectors **y**_*it*_ and **y**_*i, t*__−1_ now contain the scores of person *i* on the *M* variables at time points *t* and *t*-1 (*t* = 1, …, *T*_*i*_). Importantly, the number of time points does not have to be equal across persons, as is shown by the subscripted *i* in *T*_*i*_. p_*ik*_ denotes an element of the *I* × *K* partition matrix. When p_*ik*_ equals 1, person *i* belongs to cluster *k*; p_*ik*_ equals 0 if this is not the case. The VAR(1) regression coefficients **c**_*k*_ and **Φ**_*k*_ differ across the clusters of persons as indicated by the subscript *k*. The same assumptions as described above need to be met for the clusterwise extension, but now also across persons. More specifically, this means that intervals between the measurements should be equal for all persons. In addition, the mean of each variable should be constant across persons. Person-mean centering can be applied if this assumption does not hold. It is also assumed that the innovation covariation matrix is equal for all participants within a cluster. Note that a similar assumption is made in case of a multilevel extension of the VAR model (Bringmann et al., 2013), which focuses on quantitative rather than qualitative differences in model parameters.

Regarding the interpretation of the clusterwise VAR(1) model, the above mentioned approaches—forecasting and computing *R*^2^—can be used to compare the clusters. It is especially interesting to see how the predictions given a specific state (i.e., a particular set of variable scores) differ across the clusters. Inspecting possible differences in *R*^2^ values may also be useful, as they indicate whether the variable scores are more predictable in one cluster than in another, and for which variables this holds.

#### Data analysis

##### Loss function

For a particular number of clusters *K*, a partition matrix and the regression coefficients of each cluster are estimated by minimizing the sum of squared prediction errors:

(8) $$\begin{array}{l} L_{K}\;=\;\sum_{i\;=\;1}^{I}\sum_{t\;=\;2}^{T_{i}}{\left({\text{y}}_{it}\;-\;{\hat{\text{y}}}_{it}\right)}^{2} \end{array}$$

where ${\hat{\text{y}}}_{it}$ represents the predicted scores of person *i* for the *M* variables at time point *t*. Note that the loss function is calculated starting from the second time point of each person onwards because an observation at a previous time point is needed to determine the residual.

##### Estimation

To fit the clusterwise model to data, we propose to use an alternating least squares (ALS) approach, consisting of four steps:

1. Obtain an initial clustering of the persons, in either a random or a rational way. Empty clusters are not allowed. In the random case, each person is randomly assigned to one of the *K* clusters, with each cluster having equal probability of being assigned to. The rational start is based on hierarchical clustering, as is often done in ALS based clustering approaches (e.g., Brusco and Cradit, 2001; Steinley, 2003; Brusco and Cradit, 2005; Wilderjans and Ceulemans, 2013). Specifically, drawing on the approach of Zheng et al. (2013), we first fit a VAR(1) model to the data of each person separately. Second, we conduct a hierarchical clustering using Ward's criterion (Ward, 1963) on the Euclidean distances between the resulting VAR(1) slopes, and retain the *K* cluster partitioning as a rational start.
2. A single VAR(1) model is fitted to the data within each cluster, using OLS. To this end, the data of all persons within the cluster are vertically concatenated, with the first observation of each person being removed as mentioned above. The latter prevents that data of one person are predicted on the basis of data of another person.
3. To update the partition matrix, the following procedure is followed for each person consecutively. First, by means of the sum of squared prediction errors it is assessed how well the VAR(1) model of each cluster fits the data of the person, and the person is assigned to the cluster for which this sum is minimal. Next, the VAR(1) models of the clusters with altered memberships (if the person is assigned to a different cluster) are re-estimated with the updated partition matrix.
4. Finally, the previous step is repeated until the clustering no longer changes, or in other words, until there is no improvement in fit anymore.

Clustering algorithms are often susceptible to ending in a local minimum instead of the global minimum. Therefore, as is commonly done in cluster analysis, we advise to run the ALS procedure multiple times, once from the rational start described in step 1, and a number of times using a random start (Steinley, 2003; Ceulemans et al., 2007), and retain the best fitting solution. The Supplementary Material contains complete MATLAB code to run the ALS algorithm to fit a clusterwise VAR(1) model.

##### Model selection

To run the ALS procedure, a number of clusters *K* has to be specified. In most cases, the number of clusters is however not known beforehand. An important criterion to select the number of clusters is the interpretability of the retained model. As checking the interpretability of many different solutions can be cumbersome, a formal model selection strategy can assist in identifying a subset of interesting models. We propose to use the CHull procedure which balances model (mis)fit and complexity (Ceulemans and Kiers, 2006; Wilderjans et al., 2013) and which has been shown to perform well in the context of different models (e.g., Ceulemans and Kiers, 2006, 2009; Schepers et al., 2008; Ceulemans et al., 2011; Lorenzo-Seva et al., 2011; Bulteel et al., 2013). CHull provides a numerical way of determining the elbow in a scree plot, in which a measure of (mis)fit (in this case, the sum of squared prediction errors) is plotted as a function of a measure of complexity (in this case, the number of clusters). More specifically, the clusterwise VAR(1) model is first fitted with the number of clusters varying from *K*_min_ to *K*_max_. Then, one looks for the *K* value that maximizes the following scree test ratio *st*, indicating that adding another cluster will hardly increase the fit of the model:

(9) $$\begin{array}{l} {st}_{k}\;=\;\frac{L_{K-1}\;-\;L_{K}}{L_{K}\;-\;L_{K\;+\;1}}. \end{array}$$

In practice, as indicated by Ceulemans and Kiers (2006), we recommend to retain the models having the largest *st*-values for further inspection regarding interpretability. Free software to apply the CHull procedure is available from http://ppw.kuleuven.be/okp/software/chull/ (Wilderjans et al., 2013).

## Simulation studies

In this section, we will present the results of two simulation studies. In a first study, we evaluate the performance of the ALS algorithm when using the correct number of clusters. To assess the performance of the proposed model selection strategy, we will use a subset of the generated data sets of the first study in a second study.

### Simulation study 1

#### Research questions

The goal of the first simulation study is to evaluate the performance of the ALS algorithm when the correct number of clusters is used. Several criteria will be evaluated: (a) the occurrence of local minima, (b) the recovery of the clustering, and (c) the recovery of the VAR(1) coefficients. The effect of six data characteristics, often manipulated in simulation studies on clustering techniques, will be examined: (a) the number of clusters, (b) the number of time points per person, (c) the number of persons, (d) the similarity of the dynamical structure of the clusters, (e) the relative sizes of the different clusters, and (f) the variance-covariance matrix of the innovations. The last factor enables to evaluate the robustness of the method to violations of the stationarity assumption.

Based on previous simulations regarding clustering techniques in general, we formulate the following hypotheses. The performance of the ALS algorithm will deteriorate when the number of clusters is larger (e.g., Brusco and Cradit, 2005), when less time points per person are available (e.g., De Roover et al., 2012b), when the number of persons is lower, when the VAR coefficients of the clusters are more similar (e.g., Heylen et al., 2016), and when the sizes of the different clusters are unequal (e.g., Steinley, 2003; Brusco and Cradit, 2005). Regarding the variance-covariance matrix of the innovations, we expect that the performance will be worse when the assumption of an equal covariance matrix for the persons will be violated.

#### Design and procedure

When generating the data, the number of variables was set to six for all data sets. The following six factors were manipulated in a complete factorial design:

1. The number of clusters, at two levels: two and four;
2. The number of time points per person *T*, at three levels: 50, 100, and 500;
3. The number of persons, at three levels: 30, 60, and 120;
4. Distance between the VAR(1) regression coefficient matrices of the clusters, at three levels: only positive cross-regressive coefficients with small size differences (i.e., highly similar clusters condition), only positive cross-regressive coefficients with relatively large differences in size between the clusters (i.e., similar clusters condition), and positive and negative cross-regressive coefficients (i.e., highly dissimilar clusters condition);
5. Relative cluster sizes, at three levels (see Milligan et al., 1983): clusters of equal size (i.e., equal sizes condition), one cluster containing 10% of the persons and the remaining persons evenly distributed over the other clusters (i.e., unequal with minority condition), and one cluster consisting of 60% of the persons and an equal assignment of the remaining persons to the other clusters (i.e., unequal with majority condition).
6. The innovation covariance matrix, at two levels: whereas the variances of the innovations were always fixed to one, the covariances were either set to 0.2 for all persons (i.e., equal innovation covariance condition), or randomly set to 0.2 for some persons and to 0.4 for the others (i.e., unequal innovation covariance condition).

For each possible combination of these factors, five replications were generated resulting in 1620 (2 × 3 × 3 × 3 × 3 × 2 × 5) unique data sets.

**Figure 1:**
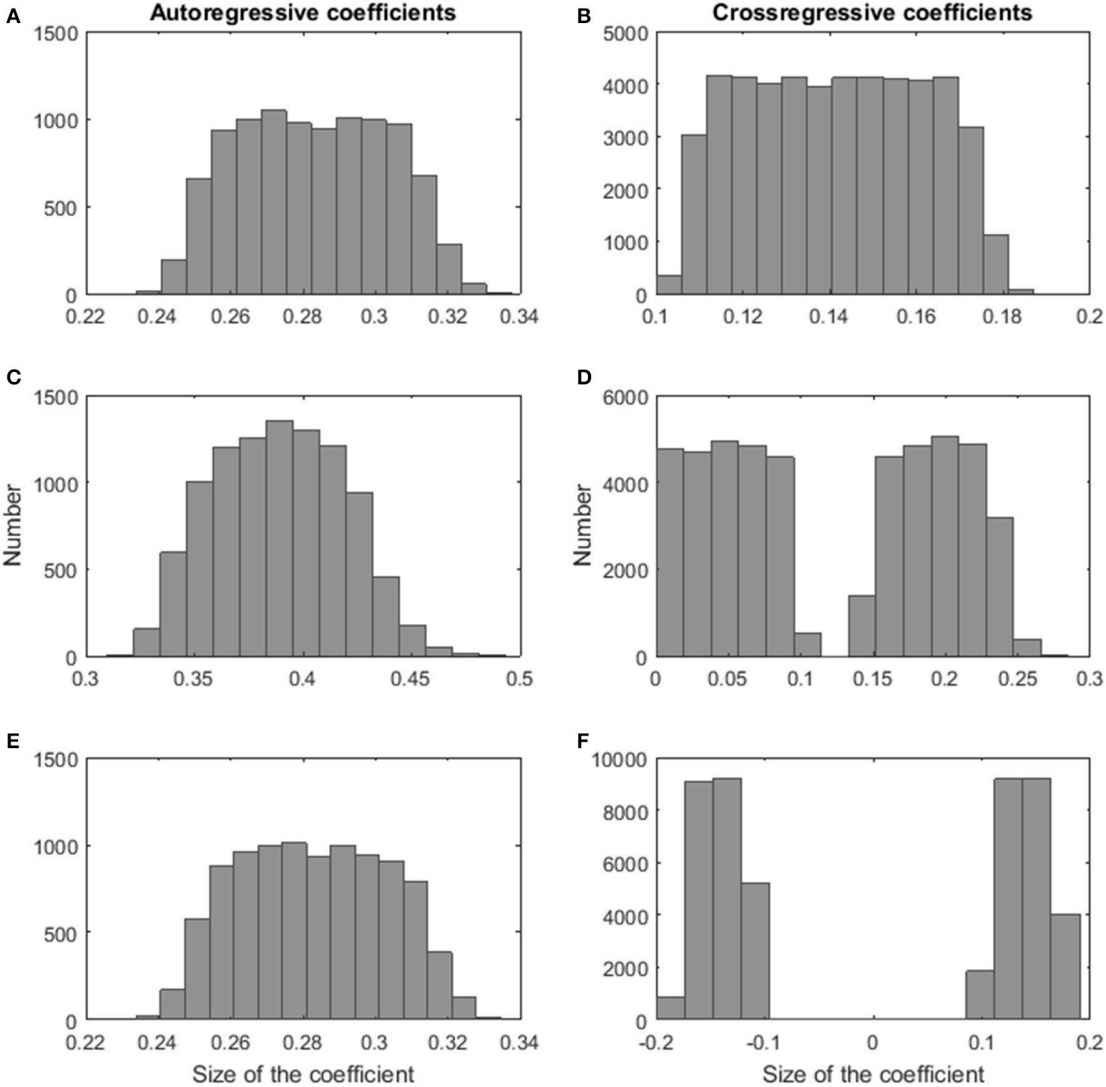
**Histograms of the size of the VAR(1) coefficients of the simulation study**. The autoregressive and the cross-regressive coefficients of the highly similar clusters condition (respectively **A**,**B**), the autoregressive and the cross-regressive coefficients of the similar clusters condition (respectively **C**,**D**), and the autoregressive and the cross-regressive coefficients of the highly dissimilar clusters condition (respectively **E**,**F**).

In particular, the next steps were executed to construct a data set. First, the number of persons per cluster was determined depending on the number of persons, the number of clusters, and on the relative sizes of the clusters. Second, a VAR(1) regression coefficients matrix was generated for each cluster. The autoregressive VAR(1) weights (i.e., the diagonal elements of the **Φ**_*k*_-matrix in Formula 7) of the clusters were drawn from a uniform distribution on the interval [0.7,0.9], whereas the cross-regressive weights (i.e., the off-diagonal elements of the **Φ**_*k*_-matrix in Formula 7) were generated conditional on the required distance between the coefficients of the clusters. In case of a highly similar dynamical structure of the clusters, we randomly sampled numbers from a uniform distribution on the interval [0.3,0.5] for each cluster. Next, we rescaled the VAR(1) regression coefficients by the following constant: $\frac{0.99}{\text{max}\left(\left|\lambda_{\Phi_{k}}\right|\right)}$, where **λ**_**Φ**_*k*__ is a vector containing the eigenvalues of **Φ**_*k*_-matrix and max refers to the maximum value of the vector. Rescaling was required to have the modulus of the largest eigenvalue smaller than 1, which is necessary to obtain stationary time series data. In the similar cluster condition, we randomly selected half of the cross-regressive coefficients for each cluster separately and drew values from a uniform distribution on the interval [0.3,0.5]. The remaining cross-regressive VAR(1) weights were generated by sampling numbers from a uniform distribution on the interval [0,0.2]. The same rescaling as in the previous condition was applied. In the highly dissimilar clusters condition, we used the same procedure as in case of highly similar clusters. Only, after rescaling the weights, we added an additional step in which we randomly selected coefficients that received a minus sign. To demonstrate the effect of the rescaling on the size of the coefficients, Figures 1A,B show histograms of the auto- and cross-regressive effects in the **Φ**_*k*_-matrices for the highly similar clusters condition, Figures 1C,D give insight into the values for the similar clusters condition, and Figures 1E,F represent the coefficients for the highly dissimilar clusters condition. The size of the values is in line with findings in psychological research (e.g., Schmitz and Skinner, 1993; Bos et al., 2012; Rosmalen et al., 2012). Third, we generated a data matrix for each person based on the VAR(1) model of the cluster the person was assigned to. A *T* × *M* matrix containing the innovations was drawn from a multivariate normal distribution with zero means and a variance-covariance matrix specified according to the sixth factor above. Starting from the innovations' values on the first time point, the data is generated according to the VAR(1) model in Formula 1. Finally, the data matrices of the persons were randomly combined to create the data set. Each data set was analyzed with the clusterwise VAR(1) algorithm, using the correct number of clusters. The algorithm was applied 101 times as 100 random starts and one rational start were used. The best solution was retained. MATLAB R2016a was used to program and run the simulation study.

#### Results

We will discuss the performance of the ALS algorithm by means of the assessment criteria introduced above. For each criterion, we will also evaluate the effect of the six manipulated factors on the performance.

##### Sensitivity to local minima

The global minimum is unknown due to the addition of innovations to the data sets. Therefore, we evaluated if the loss function value (i.e., the sum of squared prediction errors) based on the true clustering is smaller than the estimated loss function value. If this is the case, the algorithm did end up in a local minimum for sure. However, that was not true for any of the simulated data sets.

**Figure 2:**
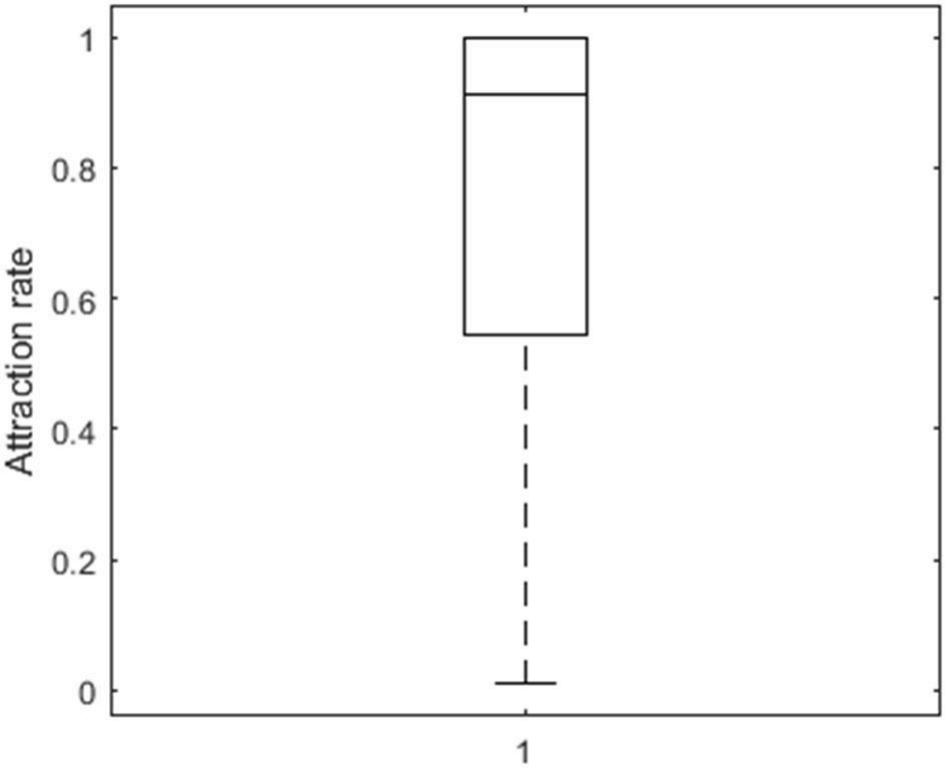
**Box plot of the attraction rate**.

**Figure 3:**
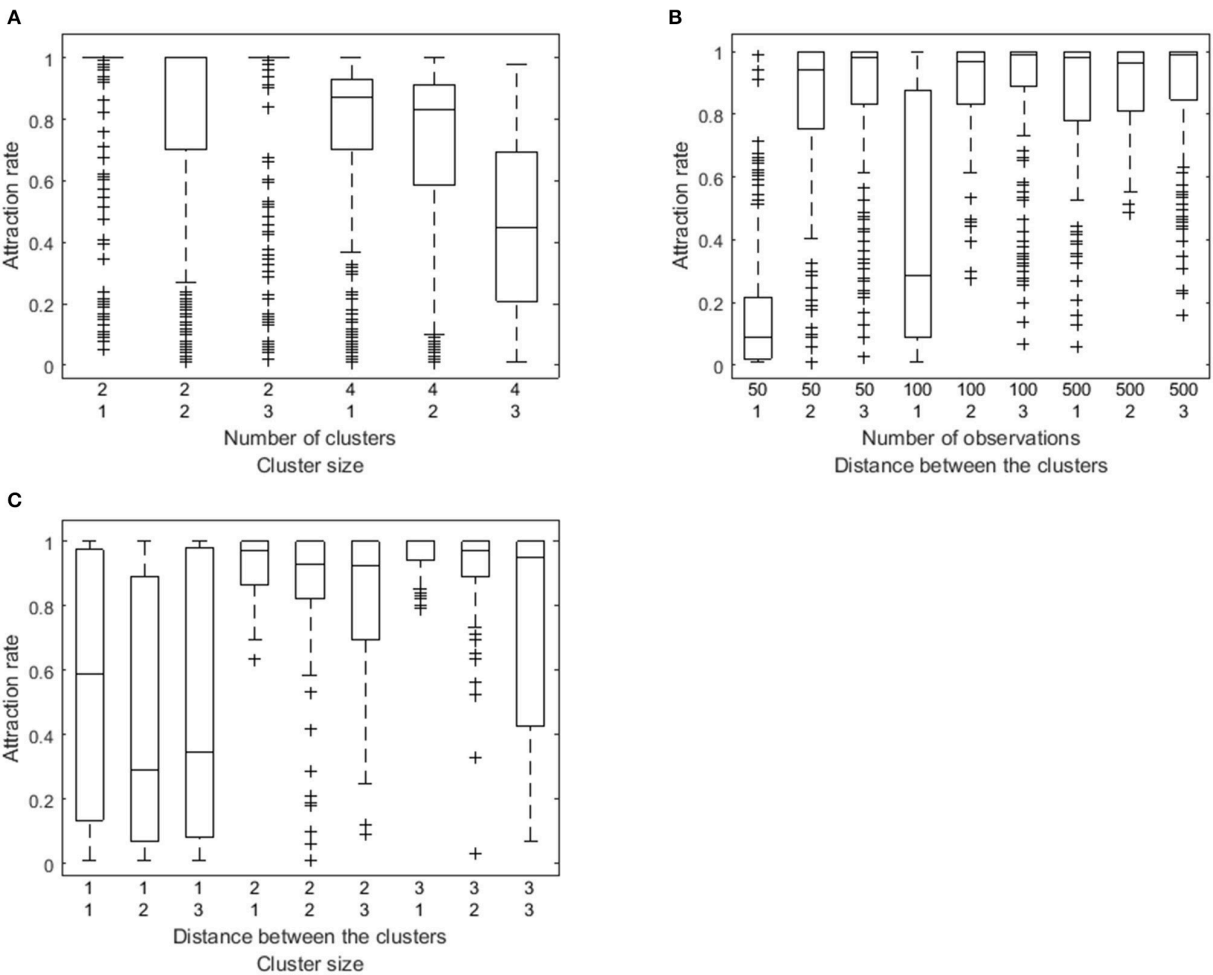
**Box plot of the attraction rate as a function of both the number of clusters and the relative cluster sizes (A), as a function of both the number of observations and the distance between the clusters (B), and as a function of the distance between the clusters and the cluster size (C)**.

To further investigate the performance of the algorithm, we computed attraction rates. Attraction rates indicate how many of the random and smart runs result in a loss function value equal to the minimum of all runs. On average, the attraction rate is 74% (*SD* = 34%) as is shown in Figure 2. A six-factorial-ANOVA, including all main and interaction effects, was performed to assess the influence of the manipulated factors. We will only focus on sizeable effects, for which the partial eta squared values ${\hat{\eta }}_{\text{p}}^{2}$ exceed 0.90. There is a substantial main effect of the number of clusters (${\hat{\eta }}_{\text{p}}^{2}=0.99$), of the number of observations per person (${\hat{\eta }}_{\text{p}}^{2}$ = 0.98), of the distance between the VAR(1) coefficients of the clusters (${\hat{\eta }}_{\text{p}}^{2}$ = 0.99), and of the relative cluster sizes clusters (${\hat{\eta }}_{\text{p}}^{2}$ = 0.95). The attraction rate is higher when the number of clusters is lower (Figure 3A), the number of observations is larger (Figure 3B), the distance between the clusters is larger (Figures 3B,C), and the cluster size is equal (Figures 3A,C). In addition, three interaction effects are substantial: between the number of clusters and the relative cluster sizes (${\hat{\eta }}_{\text{p}}^{2}$ = 0.96), between the number of time points per person and the distance between the clusters (${\hat{\eta }}_{\text{p}}^{2}$ = 0.99), and between the distance between the clusters and the relative cluster sizes (${\hat{\eta }}_{\text{p}}^{2}$ = 0.99). A smaller number of clusters alleviates the detrimental effect of unequal clusters (Figure 3A). The smaller the distance between the clusters, the larger the benefit of having more time points per person (Figure 3B). As is shown in Figure 3C, the relative cluster sizes matter more when the VAR(1) coefficients of the clusters are less similar.

##### Recovery of the clustering

To assess the accuracy of the classification, we calculate the adjusted Rand Index (*ARI*; Hubert and Arabie, 1985) between the true partition and the estimated partition. A value of 1 indicates a perfect recovery, and a value of 0 implies that the similarities of two partitions can be expected by chance. The *ARI* becomes negative if there is less overlap than at chance level. Overall, we find perfect recovery for 1211 out of the 1620 data sets (75%). The mean *ARI* equals 0.84 (*SD* = 0.33). To compare, applying only hierarchical clustering, perfect recovery was obtained for 696 data sets (43%). The mean *ARI* equals 0.61 (*SD* = 0.44) in this case.

**Figure 4:**
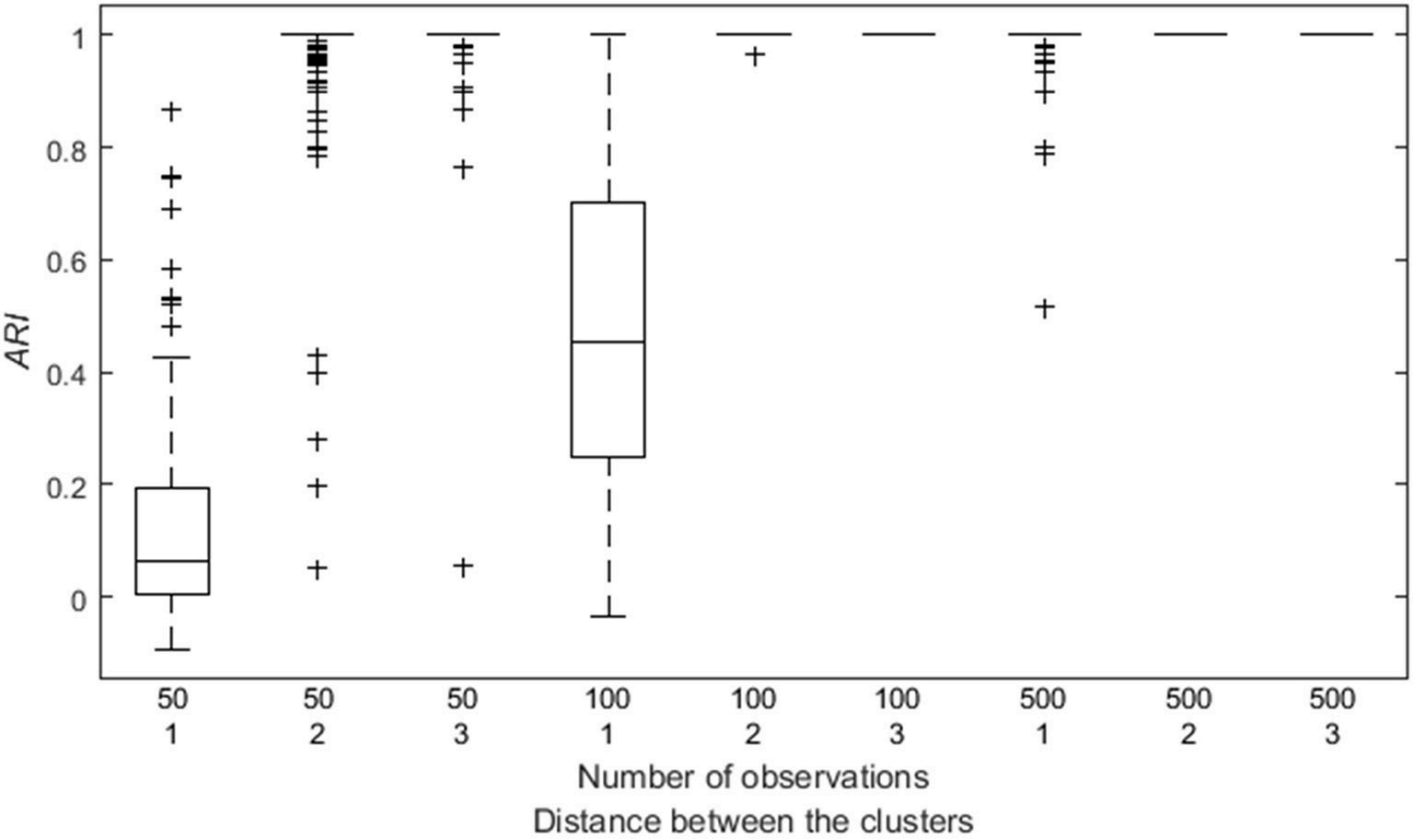
**Box plot of the *ARI* as a function of both the number of observations and the distance between the clusters**.

By means of an analysis of variance, we identified the impact of different data characteristics on the *ARI*. The main effects of the number of time points (${\hat{\eta }}_{\text{p}}^{2}$ = 0.99), and the distance between the clusters (${\hat{\eta }}_{\text{p}}^{2}$ = 1.00) are substantial. As expected, having a larger number of time points per participant, or a larger distance between the VAR(1) regression coefficient matrices of the clusters, improves the classification of the participants (see Figure 4). An interaction effect between these two factors is also present (${\hat{\eta }}_{\text{p}}^{2}$ = 1.00) indicating that having more time points per participants reduces the adverse effect of a smaller distance between the clusters, as shown in Figure 4.

##### Recovery of the coefficients

The Euclidean distance between the VAR(1) regression coefficients based on the true cluster partitioning and the estimated VAR(1) coefficients is computed to assess the recovery of the coefficients (after appropriate permutation of the estimated clusters toward the true ones). The mean Euclidean distance equals 0.16 (*SD* = 0.10).

**Figure 5:**
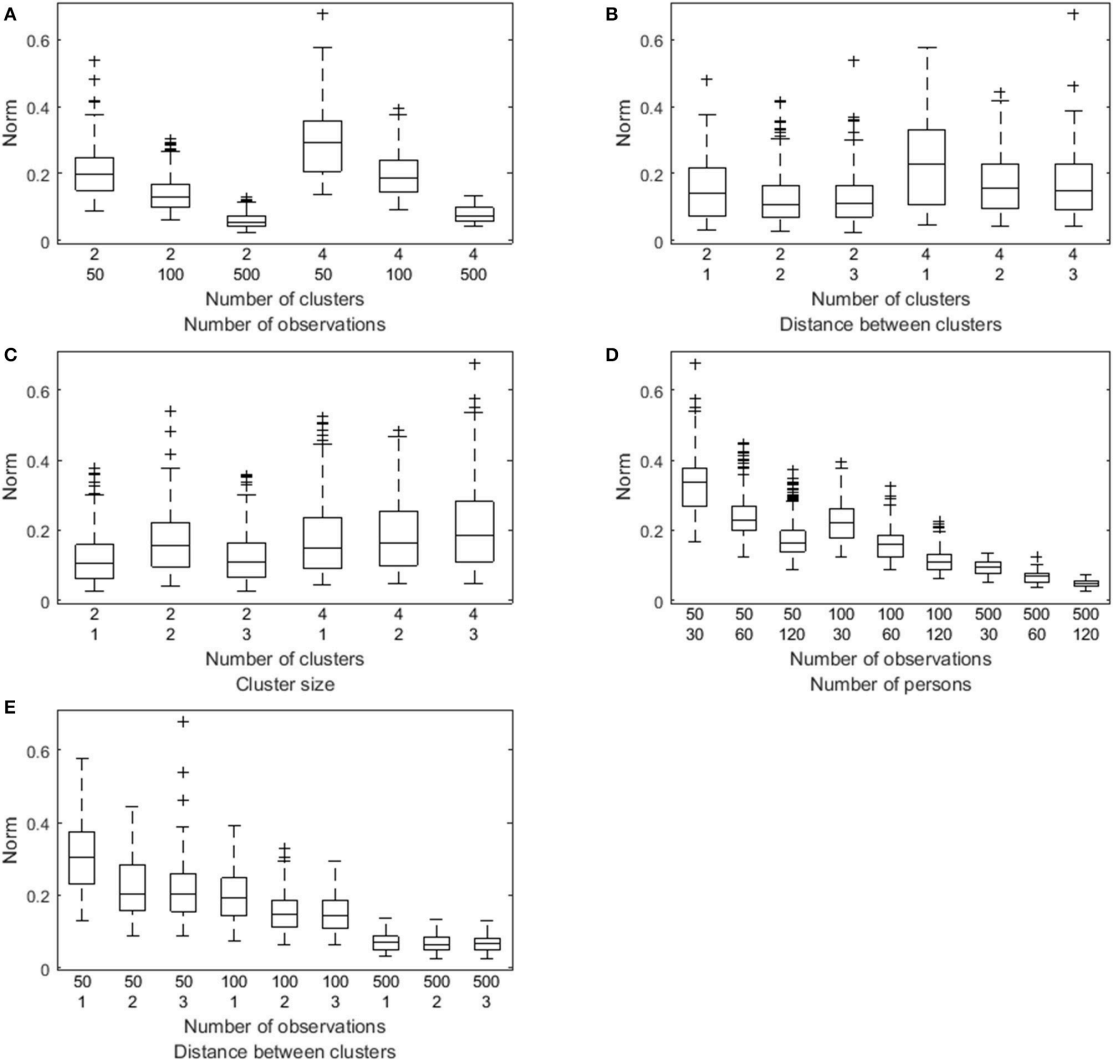
**Box plot of the Euclidian distance (denoted Norm) between the true and the estimated VAR(1) coefficients as a function of both the number of clusters and the number of observations (A), of both the number of clusters and the distance between the clusters (B), of both the number of clusters and the relative cluster sizes (C), of both the number of time points per person and the number of persons (D), and of both the number of observations per person and the distance between the clusters (E)**.

A factorial analysis of variance performed on these Euclidean distances revealed large main effects for all the manipulated data characteristics except for the innovation covariance matrix: The number of clusters (${\hat{\eta }}_{\text{p}}^{2}\;=$ 0.99), the number of observations per person (${\hat{\eta }}_{\text{p}}^{2}$ = 1.00), the number of persons (${\hat{\eta }}_{\text{p}}^{2}$ = 1.00), the distance between the VAR(1) coefficients of the clusters (${\hat{\eta }}_{\text{p}}^{2}$ = 0.99), and the relative cluster sizes (${\hat{\eta }}_{\text{p}}^{2}$ = 0.96). The VAR(1) regression coefficients are recovered worse in case of more clusters, a lower number of time points per person, a lower number of persons, a smaller distance between the clusters, or unequal cluster sizes (especially for the unequal with minority condition), as is shown by the box plots in Figure 5. Substantial interaction effects were identified between the number of clusters and the number of observations (${\hat{\eta }}_{\text{p}}^{2}$ = 0.97), between the number of clusters and the distance between the VAR(1) coefficients of the clusters (${\hat{\eta }}_{\text{p}}^{2}\;=$ 0.92), between the number of clusters and the relative cluster sizes (${\hat{\eta }}_{\text{p}}^{2}\;=$ 0.97), between the number of observations per person and the number of persons (${\hat{\eta }}_{\text{p}}^{2}\;=$ 0.98), and between the number of observations and the distance between the clusters (${\hat{\eta }}_{\text{p}}^{2}$ = 0.98). A smaller number of clusters enlarges the positive effect of a larger number of observations per persons (Figure 5A), and the positive effect of a larger distance between the clusters (Figure 5B). When the number of clusters equals two, the equal with minority conditions is clearly the worse, whereas the unequal with majority is worse when having four clusters. The equal sizes conditions results in the best recovery of the coefficients (Figure 5C). A larger number of observations slightly reduces the negative effect of a smaller number of persons (Figure 5D), but more clearly alleviates the adverse influence of a smaller distance between the clusters (Figure 5E).

### Simulation study 2

#### Research questions

The objective of the second simulation study is to assess the performance of CHull as model selection procedure. To this end, we used a subsample of the first simulation study. Because manipulating the innovation covariance matrix did not have any large effect in the first simulation study, we fixed the covariance matrix to that of the equal innovation covariance condition. For the remaining five data characteristics, we studied one of the five replications per cell of the design.

Moreover, we formulate the same hypotheses regarding the effect of the manipulated factors as for the performance of the ALS algorithm as such, because we assume that complexities in the data will not only affect model estimation but also model selection. In particular, we expect that the performance of CHull will deteriorate when the data contain more clusters, when less time points per person are available, when fewer persons are included, when the VAR coefficients of the clusters are more similar, and when the cluster sizes are not equal. For the data characteristics that were already manipulated in previous simulations investigating the performance of CHull (e.g., Schepers et al., 2008; De Roover et al., 2012a), these hypotheses were indeed confirmed.

#### Design and procedure

For each of the 162 (2 × 3 × 3 × 3 × 3) data sets retained, a clusterwise VAR(1) model was fitted with the number of clusters ranging from 1 to 6. As was the case in the first simulation study, 100 random and 1 rational starts were used to initiate each analysis. The CHull procedure was then used to determine the optimal number of clusters.

#### Results

The CHull procedure performed reasonably well, as for 78% of the simulated data sets the correct number of clusters was indicated (126 out of 162 data sets). When the number of clusters is selected correctly, the cluster assignment of the persons is in most cases perfect. The mean *ARI* for the 126 data sets equals 0.96 (*SD* = 0.17). Examining the data sets for which an incorrect number of clusters was chosen, too few clusters (two or three) are selected when the true number is four, and three, four or five clusters when the true number of clusters equals two. In some cases an additional group in between the true clusters is formed, containing elements of the different true clusters. In other cases, a pair of true clusters is merged, or exactly the opposite, one true cluster is distributed across different groups. Sometimes the obtained cluster assignment is not clearly related to the true underlying clustering.

Examining the characteristics of the 36 data sets for which the number of clusters was not correctly identified, it mainly concerns data sets having 4 clusters (29 data sets—81%), a smaller number of time points per person (for 50 time points per person: 22 data sets—61%; for 100 time points per person: 11 data sets—31%; for 500 time points per person: 3 data set—8%), a smaller number of persons (for 30 persons: 17 data sets—47%; for 60 persons: 12 data sets—33%; for 120 persons: 7 data sets—19%), and a smaller distance between the clusters (highly similar cluster condition: 27 data sets—75%; similar cluster condition: 6 data sets—17%; highly dissimilar cluster condition: 3 data sets—8%). The influence of the relative cluster sizes is less clear: 9 data sets belong to the equal sizes condition (25%), 13 data sets to the unequal with minority condition (36%), and 14 data sets to the unequal with majority condition (39%).

### Conclusion

The first simulation study demonstrated a good performance of the ALS procedure, given the correct number of clusters. Initializing the procedure with 100 random starts and 1 rational start proved more than sufficient to avoid ending in a local minimum. In line with our hypotheses, the performance is worse in more difficult conditions. All manipulated factors except for the innovation covariance matrix affected at least one of the assessment criteria. Especially too few observations per person and a small distance between the clusters were detrimental. Manipulating the variance-covariance matrix of the innovations hardly influenced the results, so the algorithm is to some extent robust to violations of the stationarity assumption. The second simulation study showed that the CHull model selection strategy performs reasonably well. Moreover, when the true number of clusters is identified correctly, the cluster assignment is almost always correct also. Again, as expected, performance deteriorated in the more difficult conditions of our design.

## Application

In this section, we apply the clusterwise VAR(1) model to time series data on depression-related symptoms of young women. This is in line with the recently proposed new view on psychiatric disorders, in which investigating the dynamics between the symptoms is considered as the key to understanding such disorders (Kendler et al., 2011; Borsboom and Cramer, 2013). The clusterwise VAR(1) model is suited to study these temporal dynamics, and in particular, to identify persons with similar dynamics. In the current application, we will analyze a subset of data from the COGITO study (Schmiedek et al., 2009). Specifically, we focus on the depression-related symptoms of the 52 younger females in the study (a VAR(1) analysis of the data of one of these women is presented in Bulteel et al., 2016)^3^. The variables were measured with 4- or 8-point Likert scales^4^. Measurements were made on about 100 close-to-daily occasions, up to six measurements a week, with a mean number of observations of 101 (*SD* = 4, ranging from 87 to 109). Depression severity was also assessed in the study, by computing the sum scores on the German version of the Center for Epidemiologic Studies Depression Scale (CES-D; Hautzinger, 1988).

To study the (co)variation of variables across time, sufficient within-person variance is by definition required (Ram et al., 2013). A first inspection of the data revealed that some of the variables had little or no variance for a number of persons. Therefore, we first discarded the variables physical symptoms, cognitive problems and restlessness, of which the scores of most participants equaled the participant modus in at least 90% of the measurement occasions (based on Ram et al., 2013). The remaining symptoms are: Rumination (8-point scale), feeling guilty (8-point scale), feeling unhappy (8-point scale), feeling downhearted (8-point scale), loss of activation (8-point scale), loss of interest (8-point scale), sleep quality (8-point scale), and loss of energy (4-point scale). Next, we removed all persons for which at least one of the remaining variables had a lack of variability, according to the 90% rule introduced above, retaining 28 participants in our analysis.

**Figure 6:**
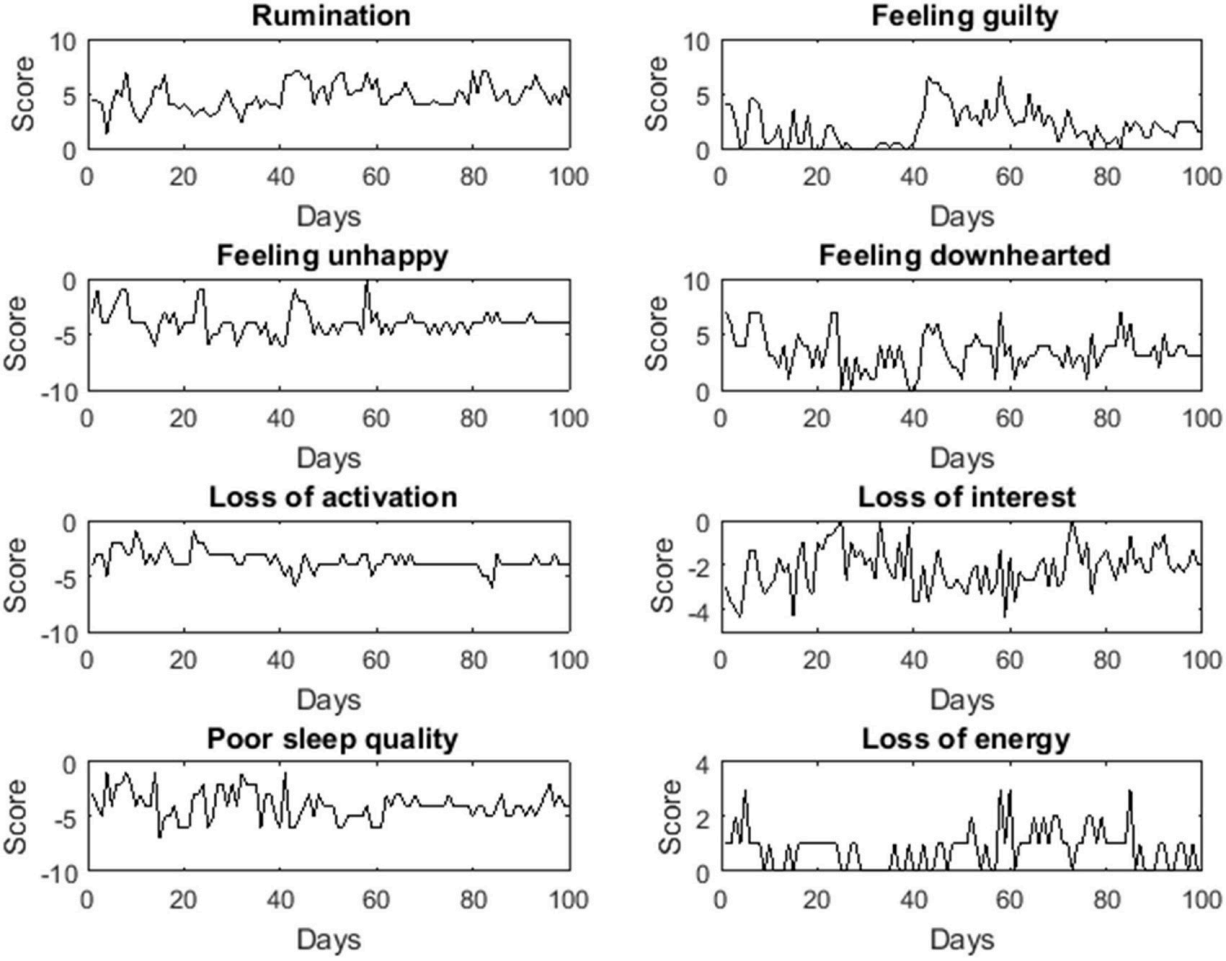
**Time series plots of the depression-related symptoms on 100 measurement occasions for one of the study participants**.

Next, we investigated whether the clusterwise VAR(1) model assumptions were met. 70% of the adjacent measurements were 1 day apart, 16% 2 days, 7% 3 days, and the remaining 7% 4 or more days. To satisfy the assumption of equidistant time intervals, we only analyzed the data with precisely 1 day between adjacent measurements. The data was further examined by means of time series plots and descriptive statistics, in order to detect clear violations of the stationarity assumption. Because the means of the depression-related symptoms differed across persons, we decided to apply the model to centered data. Figure 6 shows the time series data (before centering) of one of the women.

**Figure 7:**
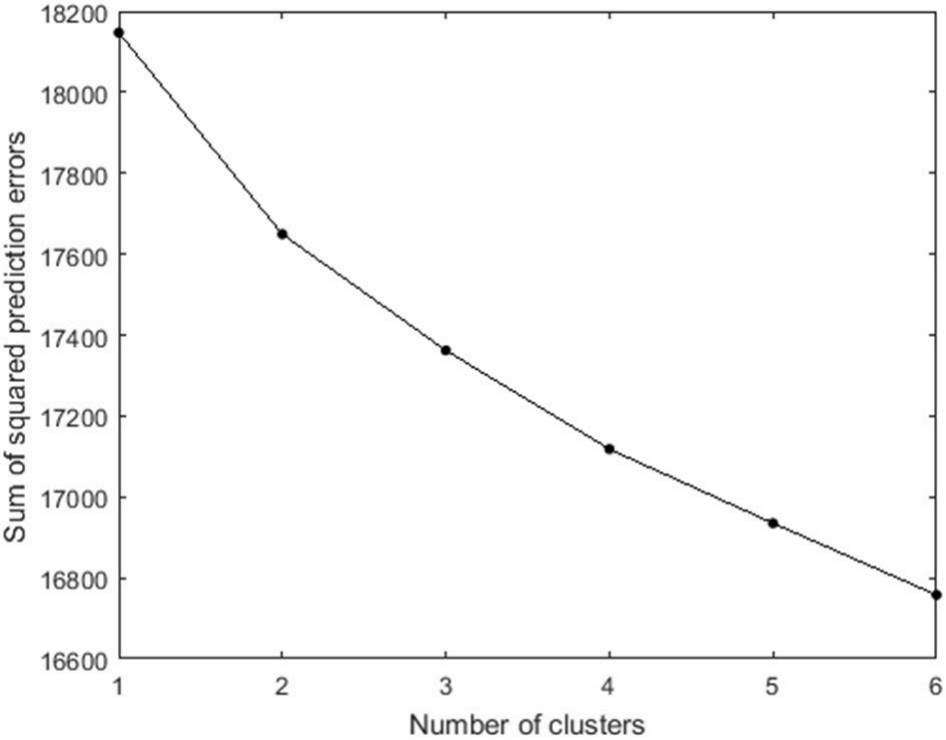
**Scree plot of the number of clusters vs. the sum of squared prediction errors for the 28 selected participants from the COGITO study**. Note that the *y*-axis does not start at 0.

Clusterwise VAR(1) analyses with 100 random starts and 1 rational start were fitted to the data, with the number of clusters ranging from one to six. Figure 7 plots the sum of squared prediction errors vs. the number of clusters. The CHull method indicates that the *st*-value is maximized for two clusters (more specifically, the *st*-values equal 1.73, 1.18, 1.33, and 1.03 for models with 2 up to 5 clusters). Thirteen participants are assigned to the first cluster, and fifteen to the second cluster.

**Figure 8:**
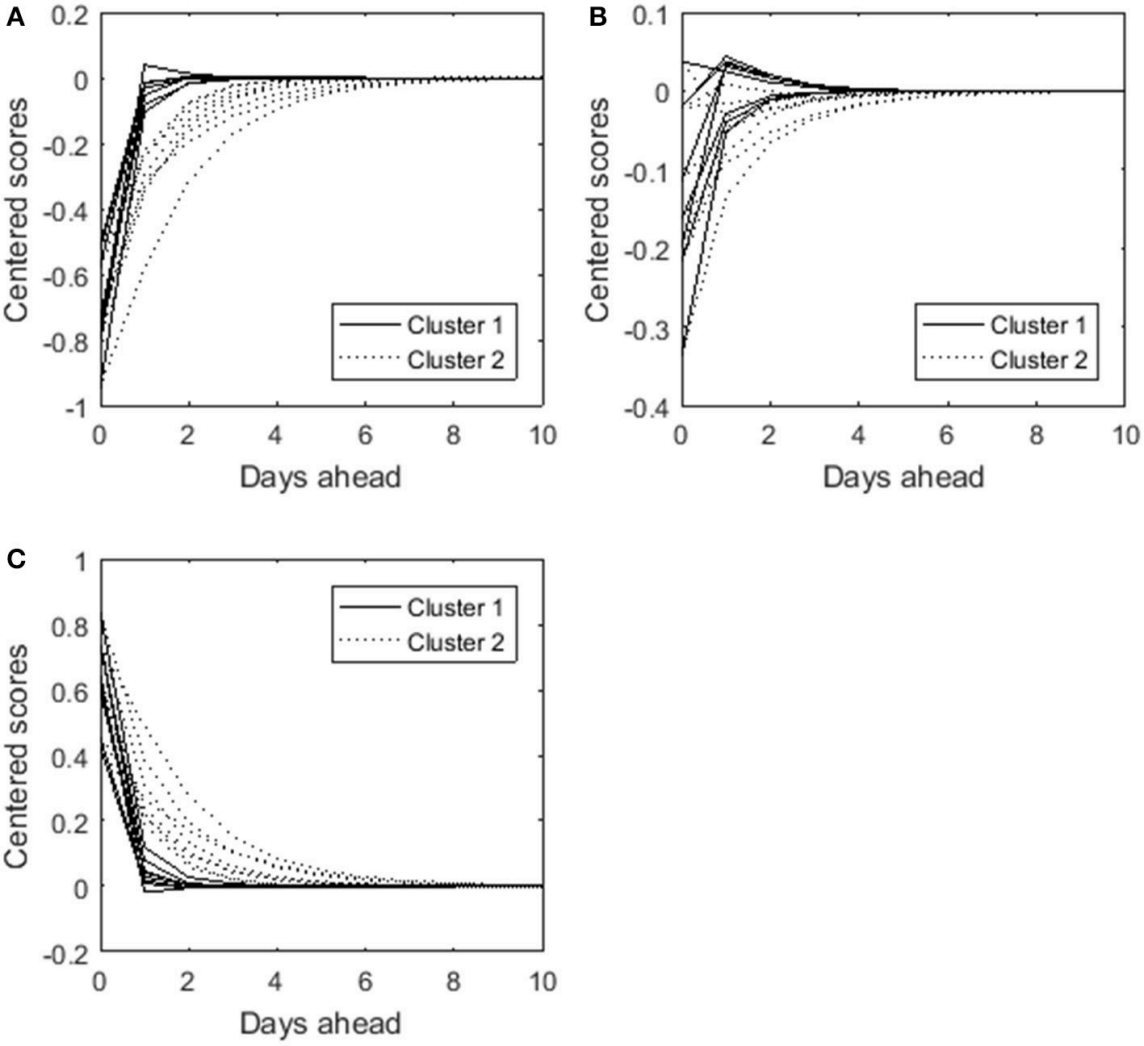
**Ten days ahead predicted scores for the eight different depression-related symptoms**. The solid lines show the predictions for the first cluster, and the dotted lines for the second cluster. The starting condition (i.e., zero days ahead) in **(A)** is that each variable score equals its first quartile, in **(B)** that each variable score equals its second quartile, and in **(C)** that each variable score equals its third quartile.

To shed light on what distinguishes both clusters we forecast the time points following a particular set of symptom scores. The symptom scores were chosen such that they reflect the most common score patterns or states (i.e., the goal was to determine realistic combinations of variable scores). To this end, we computed the quartiles of each variable. Each score was then recoded as follows: code 1 if the original score was lower than the first quartile of that variable, 2 if the score was between the first and the second quartile, 3 for the scores situated between the second and the third quartile, and 4 for scores higher than the third quartile. Examining the frequency of the coded score patterns, it appeared that having relatively low scores for (almost) all variables (i.e., lower than the first quartile, thus code 1) or having relatively large scores for (almost) all variables (i.e., higher than the third quartile, thus code 4) were the most common states. As a proxy on the original scale for these states, we used all first quartiles and all third quartiles respectively as the variable scores. Starting from these two conditions, we calculated the 10 days ahead predictions showing how the dynamic interplay influences the course of each variable for both clusters (see Figures 8A,C). Obviously, the predicted scores for both clusters eventually equal the mean value (i.e., 0 in case of centered data). The difference between the clusters lies in the persistence of the influence of the initial state. The effect lingers on for Cluster 2, whereas the influence more rapidly disappears for Cluster 1. This means for instance that a person of Cluster 2 who feels guilty at a specific point in time is more likely to indicate feeling guilty at the next occasion as well compared to a person of Cluster 1. Cluster 2 thus seems to be more resistant to change. For completeness, we added the predictions starting from the second quartiles as scores for the variables in Figure 8B. It confirms that persons in the second cluster need more time to return to their baseline (i.e., the mean values). In other words, the initial state more strongly predicts subsequent scores. Persons of the first cluster “recover” more quickly.

**Figure 9:**
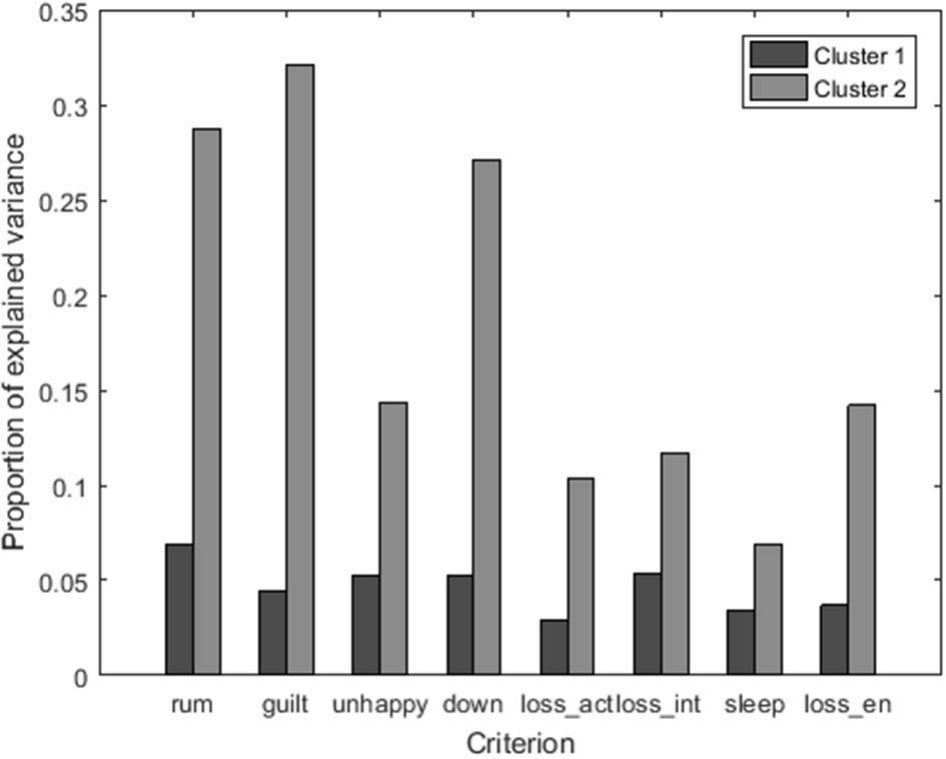
**Bar plot of the proportion of explained variance *R*^2^ for each of the eight symptoms, using the following labels: rum, rumination; guilt, feeling guilty; unhappy, feeling unhappy; down, feeling downhearted; loss_act, loss of activation; loss_int, loss of interest; sleep, sleep quality; and loss_en, loss of energy**.

In addition, the *R*^2^ for each depression-related symptom as criterion variable is shown per cluster in Figure 9. A high *R*^2^ implies that the variable scores at the previous time point determine to a large extent our predictions. In line with the findings above, the *R*^2^ is consistently higher for the second cluster. It thus seems likely that persons in the second cluster can get more stuck in the depression-related symptoms. Therefore, the first cluster can be considered as the group of “flexible responders,” and the second cluster as a group of “rigid responders.” In accordance with recent findings in the literature that revealed the role of an inert affective system for depression one can think of these persons as being at risk (e.g., van de Leemput et al., 2014).

To validate the clustering, we examined the relation between the obtained partitioning of the women and the CES-D sum scores. We conducted a nonparametric Mann–Whitney U test to examine if the participants of one of the clusters tend to have higher depression scores. This was the case for the group of “rigid responders” indeed (*z* = 2.05, *p* = 0.04).

In summary, the application of the novel clusterwise VAR(1) approach to real data yielded a meaningful result. A sample of young women were clustered in accordance with their dynamic patterns of depressive symptoms, and the clustering solution seems to reveal important individual differences of women belonging to either cluster. Moreover, the obtained partitioning of the women was related to their depression scores.

## Discussion

In this paper, we have proposed a new method to simultaneously cluster persons according to their VAR(1) regression coefficients, and fit a shared VAR(1) model to all persons within a cluster. A first benefit of our approach is that, in contrast to multilevel VAR(1) models (Bringmann et al., 2013), it captures qualitative rather than quantitative differences. Second, unlike sequential approaches (e.g., Zheng et al., 2013), our method optimizes one single loss function and pools, while updating the VAR(1) parameters, the data of all the persons within a cluster, which is known to improve the estimation of the VAR(1) parameters (Frühwirth-Schnatter and Kaufmann, 2008). Finally, in contrast to AR based techniques, our method is able to take multivariate dynamical relations into account.

The performance of the estimation procedure was assessed in a simulation study, showing good results for both recovery of the clustering and the coefficients. The results of a second simulation study revealed that the CHull model selection strategy performs reasonably well in indicating the appropriate number of clusters.

The empirical data example showed how different temporal dynamics between depression-related symptoms could be found for a group of young women. In particular, two clusters were identified. One cluster, containing about half of the women, returned fast to their mean values and were therefore labeled as the group of “flexible responders.” For the second cluster, which contained the remaining participants, the influence of what happened on the previous time points lingers; these women where called the group of “rigid responders.”

Several directions for future work can be identified. First, our current implementation of the clusterwise VAR(1) model cannot handle missing data. As our method does not require an equal amount of observations per person, we made use of listwise deletion in the application, as is mostly done in case of time series data. Other approaches for dealing with missing data are described and evaluated in Liu and Molenaar (2014). However, these methods either yield biased results, or require a large subset of complete data. Future research on this topic is thus recommended.

Second, it is straightforward to apply the proposed clusterwise extension to VAR models of an order higher than one, as long as the models of the different clusters have the same order. The development of an extension that would allow to end up with a VAR model of a different order for each cluster, would be more interesting however. It is for instance conceivable that measurements up to the second lag predict the current scores for one particular cluster, whereas a VAR model of order zero (i.e., without lagged variables) is sufficient for another cluster as no temporal dynamics are present for this group.

Third, if assumptions can be made regarding the size and the direction of the contemporaneous effects, the procedure can be extended to SVAR models as well. We would like to point to the exploratory SVAR based clustering method developed by Gates et al. (2014). In contrast to our ALS approach minimizing a single loss function, it concerns a stepwise procedure which however does not require to specify a priori hypotheses about the instantaneous relations between the variables. It would be interesting to compare the performance of both methods.

Fourth, though computationally demanding, mixture extensions of multilevel VAR could be an interesting direction for future work, because such an extension would allow to capture quantitative as well qualitative as differences. For univariate AR models, a Bayesian approach to estimate a clusterwise multilevel AR model was proposed by Frühwirth-Schnatter and Kaufmann (2008). Also in the context of standard (i.e., independent observations rather than time series data) multilevel regression models, mixture variants, were developed (Verbeke and Lesaffre, 1996; Proust and Jacqmin-Gadda, 2005). These approaches are not without difficulties, however, as the algorithms may fail to converge, if initial values are specified incorrectly (Proust and Jacqmin-Gadda, 2005).

Furthermore, a VAR model does not take measurement error into account. Instead, the stochastic part of the model consists of innovations. The influence of these innovations goes beyond a particular moment in time. By contrast, measurement error is specific to a measurement occasion (e.g., accidently indicating a wrong answer on a questionnaire). Future research could extend the clusterwise VAR model to include this type of error. A discussion on this topic and examples of autoregressive models taking measurement error into account is presented in Schuurman et al. (2015).

Finally, the clusterwise VAR(1) model could not only identify persons with similar dynamics, but also assist clinicians in assigning a tailored treatment. For now, the predictions as such offer no indications on when or how to treat persons, however. Further examining the possibilities and potential of the approach for clinical practice is recommended.

In conclusion, studies in psychology increasingly investigate within-person processes. To parsimoniously capture qualitative differences in these intraindividual dynamics, we presented a clusterwise extension of the frequently used VAR(1) model.

## Author contributions

KB, FT, and EC conceived and designed the experiments. KB conducted the simulations. KB, AB, and EC analyzed the data. KB, FT, AB, and EC wrote the manuscript.

### Conflict of interest statement

The authors declare that the research was conducted in the absence of any commercial or financial relationships that could be construed as a potential conflict of interest.

## Footnotes

^1^Mixture VAR models were proposed (e.g., Fong et al., 2007) for dealing with non-stationarity issues in one multivariate time series. These models impose a mixture distribution upon the innovations, and hence, cluster time points of the time series. Our aim is to group persons with similar stationary dynamic processes.

^2^Next to forecasting, structural analyses to investigate the precise nature of the system dynamics are possible (e.g., calculating impulse response functions). However, these methods require identification assumptions about the contemporaneous relations between the variables (see Brandt and Williams, 2007), and are therefore not considered in the current paper.

^3^We selected the younger subgroup, because for the older subgroup in this sample negative affect hardly occurs (Brose et al., 2015), which is an important constituent of depression. As explained, our technique requires sufficient variance of the variables involved to yield meaningful results.

^4^In the social and behavioral sciences, variables measured on an ordinal scale are often treated as if they were measured on a continuous scale. In the context of other models, it was already demonstrated that this assumption is acceptable in case of sufficient categories (i.e., at least five to seven; e.g., Muthén and Kaplan, 1985; Mîndrilã, 2010; Rhemtulla et al., 2012).
