# Supplementary material for: Clustering Vector Autoregressive Models: Capturing Qualitative Differences in Within-Person Dynamics
Source: Front Psychol. 2016 Oct 7;7:1540. doi: 10.3389/fpsyg.2016.01540 (PMC5054011; doi:10.3389/fpsyg.2016.01540)
Supplement: Supplementary file 1 [file DataSheet1.DOCX]

Supplementary Material

Clustering Vector Autoregressive Models: Capturing Qualitative Differences In Within-Person Dynamics

Kirsten Bulteel*, Francis Tuerlinckx, Annette Brose, Eva Ceulemans

*** Correspondence:** Kirsten Bulteel: kirsten.bulteel@kuleuven.be

# Supplementary Code

MATLAB code to estimate the clusterwise VAR(1) model by means of an alternating least squares (ALS) algorithm.

function [Pmr,Pmrvec,RegrCoeff,Fit,GOF,varDr,Y_pred]=FitClVAR1model(X,Y,nObs_per_pers,nClusters,nRandomStarts,SmartStart)

%{

This function fits a clusterwise VAR(1) model to the data by using an alternating least squares (ALS) algorithm.

INPUT

X: Lagged predictors (the total number of observations x the number of

variables). Observations belonging to the same person are a set

of consecutive rows.

An example:

[X1_time1_person1 X2_time1_person1 X3_time1_person1;

X1_time2_person1 X2_time2_person1 X3_time2_person1;

...

X1_time100_person1 X2_time100_person1 X3_time100_person1;

X1_time1_person2 X2_time1_person2 X3_time1_person2;

...

X1_time50_person2 X2_time50_person2 X3_time50_person2;

X1_time1_person3 X2_time1_person3 X3_time1_person3;

...

X1_time500_person3 X2_time500_person3 X3_time500_person3]

Y: Outcome variables (the total number of observations x the number of

outcome variables). Observations belonging to the same person are a set of

consecutive rows. The number of rows for the X- and Y-matrix

should be equal.

Back to the example:

[X1_time2_person1 X2_time2_person1 X3_time2_person1;

X1_time3_person1 X2_time3_person1 X3_time3_person1;

...

X1_time101_person1 X2_time101_person1 X3_time101_person1;

X1_time2_person2 X2_time2_person2 X3_time2_person2;

...

X1_time51_person2 X2_time51_person2 X3_time51_person2;

X1_time2_person3 X2_time2_person3 X3_time2_person3;

...

X1_time501_person3 X2_time501_person3 X3_time501_person3]

nObs_per_pers: Column vector (length = the number of persons) in which

the number of observations for each person is listed.

Returning to the example:

[nObs_person1;nObs_person2;nObs_person3]

-> [100; 50; 500]

nClusters: The number of clusters

nRandomStarts: The number of random starts

SmartStart: Binary variable: a '1' indicates the use of hierarchical

clustering as a smart start, a '0' indicates no use of a smart start

OUTPUT

Pmr: nPersons x nClusters partition matrix

Pmrvec: Partition matrix in vector format

RegrCoeff: Matrices for each cluster containing the clusterspecific

intercepts and slopes, horizontally concenated

Fit: Sum of squared prediction errors

varDr: Total sum of squares

Y_pred: Predicted values for the outcome variables

%}

%% The main function

nPersons = length(nObs_per_pers); % The number of persons

nIndepVar = size(X,2); % The number of independent variables

nDepVar = size(Y,2); % The number of dependent variables

nObs = sum(nObs_per_pers); % The number of observations

% Preallocate the output matrices

allFit = zeros((nRandomStarts + SmartStart),1);

Pmr = zeros(nPersons,nClusters,(nRandomStarts + SmartStart));

RegrCoeff = zeros(nIndepVar+1,nClusters*nDepVar,(nRandomStarts + SmartStart));

GOF = zeros((nRandomStarts + SmartStart),1);

Y_pred = zeros(nObs,nDepVar,(nRandomStarts + SmartStart));

% Calculate the total sum of squares

varDr = ssq(Y-mean(mean(Y)));

% If the number of clusters equals one, an ordinary least squares instead of an alternating least squares (ALS) algorithm is sufficient.

if nClusters == 1

Pmr = ones(nPersons,1);

Pmrvec = Pmr; % The partition matrix in vector format

[Fit,RegrCoeff,Y_pred]=CalculateFit(X,Y,Pmrvec,nObs_per_pers);

GOF=1-Fit/(varDr);

% Perform the ALS algorithm

else

% Using random starts

for run = 1 : nRandomStarts

[nClusters run]

[Pmr_run,NewFit]=CreateInitialSolution(X,Y,nObs_per_pers,nClusters);

diff=1;

% Keep updating as long as CurFit really improves

while (diff>0.000001)

CurFitTotal = NewFit;

[NewFit,RegrCoeff_run,Pmr_run,Y_pred_run] = upd_Pmr(X,Y,nObs_per_pers,Pmr_run);

diff = CurFitTotal-NewFit;

end;

GOF_run=1-NewFit/(varDr);

allFit(run) = NewFit;

Pmr(:,:,run)=Pmr_run;

RegrCoeff(:,:,run)=RegrCoeff_run;

GOF(run) = GOF_run;

Y_pred(:,:,run)=Y_pred_run;

end

% Using a rational start

if SmartStart == 1

% Indicate to which person each observation belongs

index = zeros(1,nObs);

index([1; cumsum(nObs_per_pers(1:end-1))+1]) = 1;

Part_obs = cumsum(index);

% Compute the parameters of a VAR(1) model per person

RegrModel_per_pers = zeros(nPersons,nIndepVar*nDepVar);

for pers = 1 : nPersons

ind_obs = find(Part_obs == pers);

X_cte = [ones(length(ind_obs),1) X(ind_obs,:)];

RegrCoeff_pers = pinv(X_cte'*X_cte)*X_cte'*Y(ind_obs,:);

RegrCoeff_pers = RegrCoeff_pers(2:end,:);

RegrModel_per_pers(pers,:) = RegrCoeff_pers(:)';

end

% Hierarchical clustering on the individual VAR(1) weights

Part_HierarchicalClustering = clusterdata(RegrModel_per_pers,'maxclust',nClusters,'linkage','ward');

% Run the ALS algorithm starting from the solution of the

% hierarchical clustering procedure

[Fit_initial,~,~] = CalculateFit(X,Y,Part_HierarchicalClustering,nObs_per_pers);

IM = eye(nClusters);

Pmr_run = IM(Part_HierarchicalClustering,:);

CurFitTotal = Fit_initial;

diff = 1;

% Keep updating as long as CurFit really improves

while (diff>0.000001)

[NewFit,RegrCoeff_run,Pmr_run,Y_pred_run] = upd_Pmr(X,Y,nObs_per_pers,Pmr_run);

diff = CurFitTotal-NewFit;

CurFitTotal = NewFit;

end;

GOF_run=1-NewFit/(varDr);

allFit(end) = NewFit;

Pmr(:,:,end)=Pmr_run;

RegrCoeff(:,:,end)=RegrCoeff_run;

GOF(end) = GOF_run;

Y_pred(:,:,end)=Y_pred_run;

end

% Select the model with the best fit

[Fit,Index_RunWithBestFit] = min(allFit);

Pmr = Pmr(:,:,Index_RunWithBestFit);

RegrCoeff = RegrCoeff(:,:,Index_RunWithBestFit);

GOF = GOF(Index_RunWithBestFit);

Y_pred = Y_pred(:,:,Index_RunWithBestFit);

Pmrvec = sum(Pmr*diag(1:nClusters),2);

end

end

%% The subfunctions

function t = ssq(a)

%SSQ SSQ(A) is the sum of squares of the elements of matrix A.

t=a(:)'*a(:);

end

function [NewFit,RegrCoeff,Y_pred]=CalculateFit(X,Y,Pmr_vec,nObs_per_pers)

% CALCULATEFIT Calculate the regression model per cluster.

nIndepVar = size(X,2);

nDepVar = size(Y,2);

nClusters = max(Pmr_vec);

nObs = sum(nObs_per_pers);

% Preallocate output matrices

RegrCoeff = zeros(nIndepVar+1,nDepVar*nClusters); % Preallocate matrix for clusterspecific intercepts & slopes

Y_pred = zeros(nObs,nDepVar); % Preallocate matrix for predicted Y scores

% Indicate cluster of each observation

index = zeros(1, nObs);

index([1; cumsum(nObs_per_pers(1:end-1))+1]) = 1;

Part_obs = Pmr_vec(cumsum(index), :);

% Calculate slope of each cluster and estimate Y

for cl=1:nClusters

ind_obs = find(Part_obs==cl);

X_cte = [ones(length(ind_obs),1) X(ind_obs,:)];

RegrCoeff_cl = pinv(X_cte'*X_cte)*X_cte'*Y(ind_obs,:);

Y_pred(ind_obs,:) = X_cte*RegrCoeff_cl;

RegrCoeff(:,(nDepVar*(cl-1)+1):cl*nDepVar) = RegrCoeff_cl;

end

NewFit = ssq(Y-Y_pred);

end

function [Pmr,Pmr_vec] = randpartition(nPersons,nClusters)

% RANDPARTITION Generate random partition matrix for the one-mode clustering problem

IM = eye(nClusters); % Possible rows of the partition matrix (the number of rows equals the number of clusters)

% Preallocate the partition matrix

Pmr = zeros(nPersons,nClusters);

% As long as there are empty clusters, search process continues

while sum(sum(Pmr)==0)>0

Pmr_vec = ceil(nClusters*rand(nPersons,1));

Pmr = IM(Pmr_vec,:);

end

end

function [Pmr,CurFit] = CreateInitialSolution(X,Y,nStim_per_pers,nClusters)

%CREATEINITIALSOLUTION This function creates an initial partition matrix

% and corresponding fit, based on a random assignment to clusters.

nPersons = size(nStim_per_pers,1);

[Pmr,Part_vec] = randpartition(nPersons,nClusters); % Distributes the persons randomly over the clusters

[CurFit]=CalculateFit(X,Y,Part_vec,nStim_per_pers);

end

function [NewFit,RegrCoeff,Pmr,Y_pred]=upd_Pmr(X,Y,nObs_per_pers,Pmr)

%UPD_PMR Update the partition matrix

nPersons = size(nObs_per_pers,1);

nClusters = size(Pmr,2);

IM = eye(nClusters); % Possible rows of the partition matrix (the number of rows equals the number of clusters).

for pers = 1:nPersons

fit_cl = zeros(nClusters,1);

for cl=1:nClusters

% Put person in every cluster (1 per 1) and recalculate the fit

Pmr(pers,:) = IM(cl,:);

Part_vec_trial = sum(Pmr*diag(1:nClusters),2);

[NewFit_trial] = CalculateFit(X,Y,Part_vec_trial,nObs_per_pers);

fit_cl(cl,1) = NewFit_trial;

end;

% Put person in cluster that gives best fit

best = find(fit_cl == min(fit_cl));

Pmr(pers,:) = IM(best(1),:);

end;

Pmr_vec = sum(Pmr*diag(1:nClusters),2);

[NewFit,RegrCoeff,Y_pred] = CalculateFit(X,Y,Pmr_vec,nObs_per_pers);

end
